# Urinary Exosomal Thyroglobulin in Thyroid Cancer Patients With Post-ablative Therapy: A New Biomarker in Thyroid Cancer

**DOI:** 10.3389/fendo.2020.00382

**Published:** 2020-06-16

**Authors:** Tse-Ying Huang, Chih-Yuan Wang, Kuen-Yuan Chen, Li-Ting Huang

**Affiliations:** ^1^Department of Internal Medicine, National Taiwan University Hospital Yun-Lin/Hsin-Chu Branch, College of Medicine, National Taiwan University, Taipei, Taiwan; ^2^Department of Surgery, National Taiwan University Hospital, College of Medicine, National Taiwan University, Taipei, Taiwan

**Keywords:** thyroid cancer, thyroglobulin, exosome, urine, galectin-3

## Abstract

**Background:** Most patients with thyroid cancer typically receive thyroidectomy with ablative radioactive iodine therapy. Such patients were followed with thyroid ultrasonography and serial serum thyroglobulin evaluation. Exosomes are nanovesicles secreted into extracellular environments, including plasma, saliva, urine, and other body fluids of patients with cancer. We try to find the early prognostic and exosomal biological markers of urine.

**Methods:** We analyzed urinary exosomal proteins, including thyroglobulin and galectin-3, to identify early prognostic biological markers in urine for patients receiving operation and radioactive iodine ablative therapy. We enrolled sixteen newly diagnosed patients with papillary thyroid carcinoma and follicular thyroid carcinoma. We collect all patient's urine samples before operation, immediately after operation, post-operatively at three and six months (4 collections per patient). The levels of pre-operative and post-ablative of U-Ex Tg and galectin-3 in patients with thyroid cancer were measured.

**Results:** Trends in urinary thyroglobulin concentrations in patients with post-ablative thyroid cancer were detected in the first sixteen patients. Importantly, serum thyroglobulin was not detected in five patients after operation and radioactive I-131 ablation, while U-Ex Tg still showed an increasing trend, which implicating the probable recurrence of thyroid cancer. This is the first study to evaluate whether U-Ex Tg is a future biological marker as a substitute for serum thyroglobulin.

**Conclusion:** Our study have developed a brand-new evaluation for tracking thyroid cancer. The most useful scenario in using a test that is potentially more sensitive than existing serological testing is to eliminate the suspicion of recurrence and remove subjects from long term follow up.

**Trial Registration:** ClinicalTrials.gov: NCT02862470; 5, August 2016. https://clinicaltrials.gov/ct2/show/NCT02862470?term=NCT02862470&rank=1.

ClinicalTrials.gov: NCT03488134; 3, August 2018. https://clinicaltrials.gov/ct2/show/NCT03488134?term=NCT03488134&draw=2&rank=1.

## Introduction

Papillary and follicular thyroid cancers are low-grade endocrine malignancies, and, the core issue of differentiated thyroid cancer is surgical approach, which remain the primary mode of therapy (1–3), but reliable post-operation follow-up biomarker has remained further discussable issue. Most patients typically receive thyroidectomy with ablative radioactive iodine therapy (4, 5). These patients are followed with thyroid ultrasonography and serial serum thyroglobulin evaluation. Serum thyroglobulin is a pivotal biomarker for detecting possible residual tumors or recurrence of thyroid cancer (4). Generally, such patients appear to have a higher residual risk of isolated thyroglobulinemia, and postoperative serum thyroglobulin suggest distant metastases (4). Low-risk patients with non-stimulated postoperative serum thyroglobulin are typically defined as having less than 0.2 ng/mL or with thyroid hormone withdrawal thyroglobulin of less than 1.0 ng/mL (6, 7). However, costly recombinant human TSH (rhTSH) is often required to stimulate serum thyroglobulin for detecting local recurrence or distant metastasis (8). Thus, earlier biological markers for predicting the prognosis of thyroid cancer are needed.

Exosomes are nanovesicles secreted into extracellular environments. Cancer cell-derived exosomes are found in the plasma, saliva, urine, and other body fluids of patients with cancer. Increasing evidence suggests that exosomes can be used as biomarkers for the diagnosis and prognosis of malignant tumors (9, 10). Exosomes are 40–100 nm in diameter and correspond to the intraluminal vesicles of endosomal multivesicular bodies (11). Exosomes secreted by cells transfer molecular messages between cells and may be useful biological markers of cancer (11). In addition, exosomes can be collected from the serum, tissue fluid, and urine for diseases follow-up, and collecting urine as a biosample is easier to repeatedly obtain and non-invasive (12, 13). In this study, we enrolled patients with papillary and follicular thyroid carcinoma, and collected their urine samples before operation, immediately after operation, and post-operatively 3 and 6 months. We analyzed the urinary exosomal proteins to identify early prognostic biological markers in the urine in this prospective study.

## Materials and Methods

### Patients

The prospective study enrolled patients with newly diagnosed thyroid cancer. This preliminary study report included sixteen patients who were followed-up for six months. We enrolled 23 patients in the very beginning, and 16 patients completed all 4-times check-ups in our study. All sixteen patients received total thyroidectomy under clinically surgical judgment, and twelve patients received radioactive I-131 ablation at approximately four weeks after operation.

### Study Design

We enrolled the newly diagnosed patients with thyroid papillary, follicular cancer. After signing inform consent, we collect their urine samples before operation, immediately after operation, post-operatively at three and six months (4 collections per patient). This prospective study was approved by (Ethical Review Board approval) Institution Responsibility Board of National Taiwan University Hospital and complete patient inform consent. We identify the committee that approved the research and confirm that all research was carried out in accordance with relevant guidelines/regulations, and informed consent was obtained from all participants.

### Peptide Sequences

Thyroglobulin sequence and galectin-3 sequence are available from Peptide Atlas (https://db.systemsbiology.net/sbeams/cgi/PeptideAtlas/GetSELExperiments) and UniProt (http://www.uniprot.org/) in Table 1. Standard peptides were synthesized by Mission Biotech, Ltd. (Taipei, Taiwan). The synthetic peptides were dried and precipitated with ether. Peptides were purified by reverse-phase high-performance liquid chromatography while monitoring peptide elution at 230 nm.

**Table 1:** Peptide standards list and representative protein.

**Peptide**	**Sequence**	**Molecular weight (Dalton)**
Thyroglobulin	FLAVQSVISGR	1176.38
Galectin-3	IALDFQR	862.00

### Urine Collection

Urinary exosome precipitation was performed. First, 200 mL of fresh human urinary sample was collected for exosome precipitation. These samples were centrifuged at 3000 × *g* for 15 min at 4°C to remove cells and cell debris, and then centrifuged at 10,000 × *g* for 30 min at 4°C to remove microvesicles. Amicon^®^ Ultra 15-centrifugal filters, 100K (Millipore, Billerica, MA, USA) were used to concentrate the 200-mL urinary samples to 5–10 mL. Urinary exosomes were isolated using ExoQuick-TC (System Biosceinces, Palo Alto, CA, USA). Supernatants were transferred to new tubes, completeTM, EDTA-free Protease Inhibitor Cocktail (Roche, Basel, Switzerland) was added, and samples were stored at−80°C. Exosome pellets were resuspended in lysis buffer (7 M urea, 2 M thiourea, 4% CHAPS). Exosome protein samples were frozen at −80°C until multiple reaction monitor (MRM) analysis.

### Reagents and Chemicals

All reagents were ACS grade or higher. All solvents used, including water, were liquid chromatography (LC)/mass spectrometry (MS) grade.

### Tryptic Digestion

Urinary exosome samples were precipitated with three volumes of cold methanol at −20°C, followed by centrifugation at 10,000 × *g* for 10 min. The pellet was then suspended in lysis buffer (4 M urea, 25 mM ammonium bicarbonate, pH 8.5). The denatured samples were reduced with 200 mM dithiothreitol at ambient temperature for 1 h and then alkylated with 200 mM iodoacetamide in the dark for 1 h. The remaining iodoacetamide was quenched by the addition of 200 mM DTT and incubated at ambient temperature for 20 min. Modified sequencing-grade trypsin (Promega, Madison, WI, USA) was added to samples. Digestion was carried out for 16 h at 37°C.

### MRM Q1/Q3 Ion Pair Selection Using Direct Infusion

Synthetic standard peptides were diluted to 2 μg/mL in 0.1% formic acid for infusion at a flow rate of 10 μL/min using a syringe pump. The infused peptide solutions were analyzed by electrospray ionization using an AB SCIEX QTRAP 5500 mass spectrometer (Framingham, MA, USA) equipped with the TurboV source and controlled by Analyst software 1.5. MS analysis was conducted in positive ion mode with the ion spray voltage set to 5500 V. The source temperature was set to 550°C. Additional parameters were nebulizer and drying gas flow at 60 and 45 psi, respectively. Analyst software (version 1.5) was used to generate a list of all possible b- and y-series fragment ions for both 2+ and 3+ precursor ion-charge state spanning *m/z* range from 100 to 1000. MRM scans for optimization of MRM Q1/Q3 ion pairs were conducted with both Q1 and Q3 set to unit resolution (0.7 Da full width at half maximum), while the collision energy (CE) was ramped from 5 to 55 V in 1-V increments, with dwell time of 150 ms for each transition. From this data, the four transitions that produced the strongest signals were selected on a per-peptide basis.

Next, the three transitions producing the most abundant signals free of signal interferences were selected from these four transitions.

### LC-MRM/MS Analysis of Urinary Exosome Digests

An Agilent 1260 Infinity HPLC system (Agilent Technologies, Santa Clara, CA, USA) was used to directly inject 10 μL of urine digest samples onto a reverse-phase analytical column (100 × 2.1 mm i.d., 2.7 μm, Agilent Poroshell 120 EC-C18) that was maintained at ambient temperature. Samples were separated using a 300 μL/min flow rate and gradient of 3–90% of mobile phase B over a total run time of 30 min. Mobile phase A consisted of 0.1% v/v formic acid, while mobile phase B consisted of ACN/0.1% formic acid. The gradient method is composed of multiple linear gradients as follows (time: %B) : 0.1 min, 10% B; 3.5 min, 11% B; 6.5 min, 20% B; 7 min, 21% B; 7.5 min, 22% B; 12.5 min, 22.5% B; 17 min, 25% B; 20 min, 30% B; 22.5 min, 42% B; 23.5 min, 90% B; 27 min, 3% B; 30 min, 3% B. An AB SCIEX QTRAP 5500 with a TurboV ionization source, controlled by Analyst software, was used for all LC-MRM/MS sample analyses. All acquisition methods used the following parameters: 5500 V ion spray voltage, nebulizer and drying gas flow of 60 and 45 psi, respectively, source temperature of 550°C, and Q1 and Q3 set to unit resolution (0.7 full width at half maximum).

MRM acquisition methods were initially composed of four ion pairs per peptide during determination of high-signal producing interference-free transitions and LC method development. The final analytical method was composed of one verified quantifier ion pair per peptide, and is a high-throughput, rapid 30-min method that has been evaluated for common urine interferences. However, urine analysis of the samples was performed with acquisition methods containing three verified ion-pair transitions per target peptide to ensure the detection of any minor sample-specific signals. MRM acquisition methods were constructed using fragment ion-specific tuned CE voltages and retention time constraints.

### MRM Data Analysis

All MRM data were processed using AB SCIEX Analyst software (version 1.5) with the Integrator algorithm for peak integration set to default values. All integrated peaks were manually inspected to ensure correct peak detection and accurate integration. Linear regression of all calibration curves was performed using a standard 1/x2 (x = concentration) weighting option to aid in covering a wide dynamic range. The concentration of each peptide target was calculated based on the observed response and experimentally determined linear regression equation from the standard curve. The calculated concentration is reported in μM of urine and can be defined in ng/mL when the weight of the entire processed protein is taken into account.

### Thyroglobulin and Anti-thyroglobulin Antibody

Thyroglobulin levels were determined using IMMULITE 2000 Thyroglobulin, a solid-phase, chemiluminescent immunometric assay. Its analytical sensitivity is 0.2 ng/mL (Siemens, Erlangen, Germany). An anti-thyroglobulin antibody survey was conducted in the ARCHITECT Anti-Tg assay, which is a two-step immunoassay for quantitative determination of thyroglobulin autoantibodies in human serum. Its sensitivity is ≤ 1.0 IU/mL (Abbott Laboratories, Chicago, IL, USA).

## Results

### Patients Demographics

All patients receive pre-operative and post-operative investigation of thyroid function and serum thyroglobulin. All demographic information with surgical pathological findings are shown in Table 2.

**Table 2:** Demography of patients before and after operation.

**Patient number**	**Gender**	**Age**	**Date of OP**.	**Date of radioactive I-131**	**I-131 Ablation Dose**	**FT4 Post-OP**.	**hsTSH Post-OP**.	**Tg Pre-OP**.	**Tg Post-OP. 6 months**	**Anti-Tg Ab Post-OP. 6 months**	**Pathology**
1	F	49	20160820	20160929	30 mCi	0.981	0.212	93.6	<0.2	<3.0	PTC with minimally extra-thyroid soft tissue involvement,
											TNM: T3N0M0
2	F	58	20160908	NA	NA	1.13	2.12	NA	<0.2	50.4	PTC with clear surgical margin,
											TNM: T1aN0M0
3	F	57	20160912	20161014	30 mCi	1.24	2.96	NA	<0.2	<3.0	PTC with minimally extra-thyroid soft tissue involvement, central lymph
											nodes dissection
											TNM: T3N0M0
4	F	42	20160912	20161014	30 mCi	1.24	0.611	<0.2	<0.2	6.03	PTC
											TNM: T1bN1aM0
5	F	34	20161015	20161118	30 mCi	1.07	11.3	NA	<0.2	<3.0	PTC with minimally capsular invasion,
											TNM: T3N0M0
6	F	52	20161119	NA	NA	1.01	0.249	NA	3.8	4.47	PTC
											TNM: T2N0M0
7	F	43	20161208	20170120	30 mCi	<0.27	233	NA	<0.2	NA	PTC
											TNM: T1bN0M0
8	M	48	20161205	20170112	30 mCi	0.372	57.7	NA	<0.2	NA	Right thyroid follicular carcinoma
											TNM: T1bN0M0
9	M	41	20170422	20170720	150 mCi	2.23	0.059	NA	27.4	3.46	Papillary carcinoma, with minimal extrathyroid extension,
											TNM: T3N0M1 (lung)
10	F	59	20170410	NA	NA	1.27	0.96	NA	9.14	<3.0	Papillary microcarcinoma
											TNM: T1aN0M0
11	F	45	20170318	20170421	30 mCi	0.963	118	NA	<0.2	<3.0	PTC (multifoci)
											TNM: T3N0M0
12	F	41	20170501	20170726	125 mCi	1.32	0.998	1.34	<0.2	119.52	PTC
											TNM: T1bN1aM0
13	M	49	20161015	NA	NA	0.999	1.51	29.4	15.9	<3.0	PTC
											TNM: T3N0M0
14	F	39	20170619	20170721	30 mCi	1.25	1.9799	46.2	<0.2	<3.0	PTC
											TNM: T1aN1aM0
15	F	74	20170828	20170929	30 mCi	1.12	0.202	14.1	<0.2	<3.0	PTC
											TNM: T1aN1aM0
16	F	31	20171005	20180208	100 mCi	1.29	1.9095	57.6	0.217	<3.0	PTC
											TNM: T3N1bM0

*T1 or T2: patients 2, 6, 7, 8, 10; T3 or lymph nodes metastasis: patients 1, 4, 5, 11, 12, 13, 14, 15, 16*;

*T3 with distant metastasis or suspect delphian lymph nodes metastasis: patients 3, 9*.

### Urinary Biomarkers

The initial quantification of U-Ex Tg and galectin-3 is shown in Supplemental Data. Peptide concentrations are shown in units of μM. We converted the unit of μM into ng/mL using the formula: (ng/mL) = (μM) (molecular weight in Dalton).

Urinary peptide biomarkers concentrations are shown in before conversion as μM and after conversion as ng/mL. Our data showed that thyroglobulin was not detected with high sensitivity (<0.2 ng/mL), but U-Ex Tg was detected by peptide sequencing. The trends in urinary thyroglobulin concentrations were recorded individually for each patient. Particularly, serum thyroglobulin was not detected in patients 1, 3, 4, and 7 after thyroidectomy and/or radioactive I-131 ablation; however, U-Ex Tg showed an increasing trend. This condition may implicit the tumor burden and the recurrence of thyroid cancer in the future.

**Figure 1:**
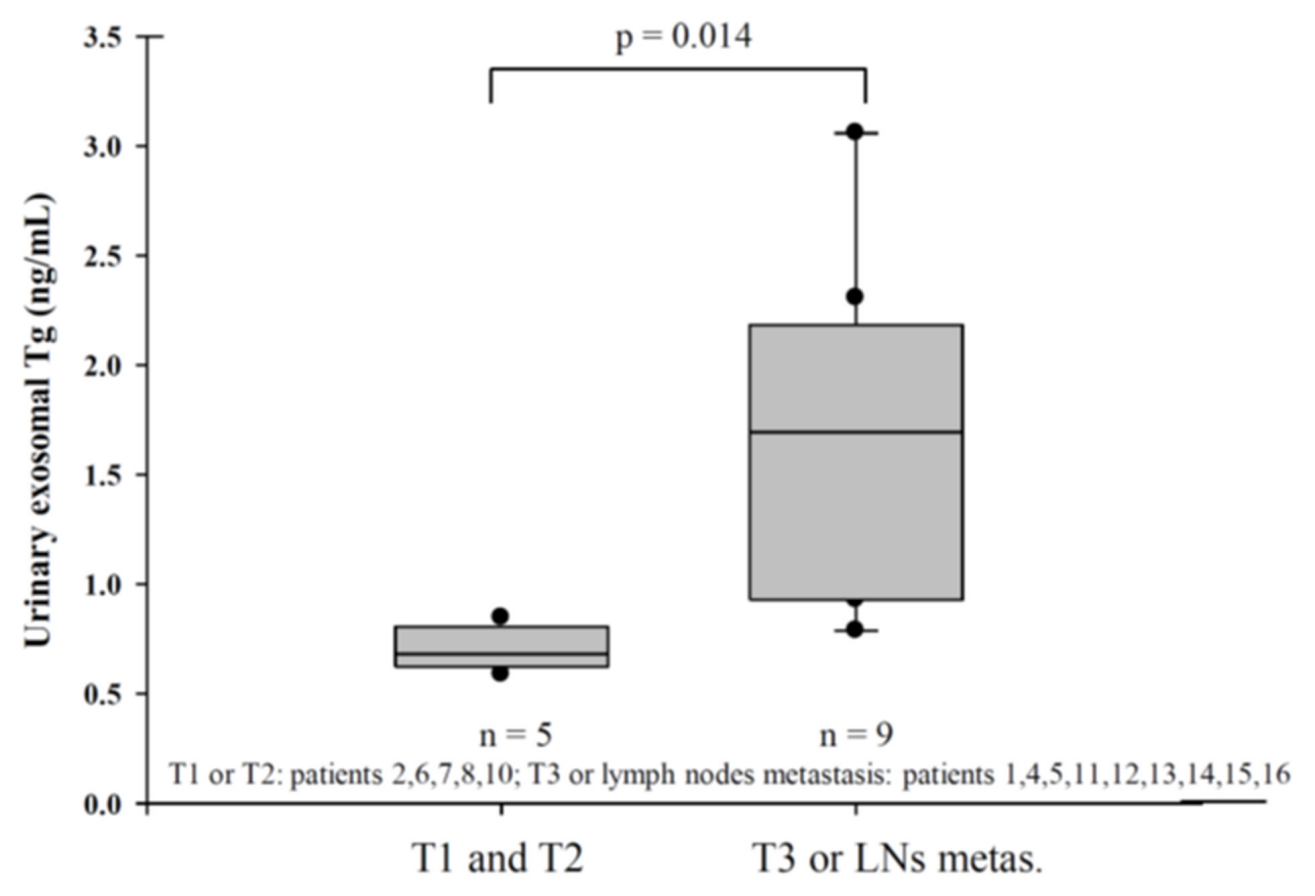
Urinary exosomal thyroglobulin revealed statistically significant in larger tumor (T3) or lymph nodes metastasis in pathological report.

**Figure 2:**
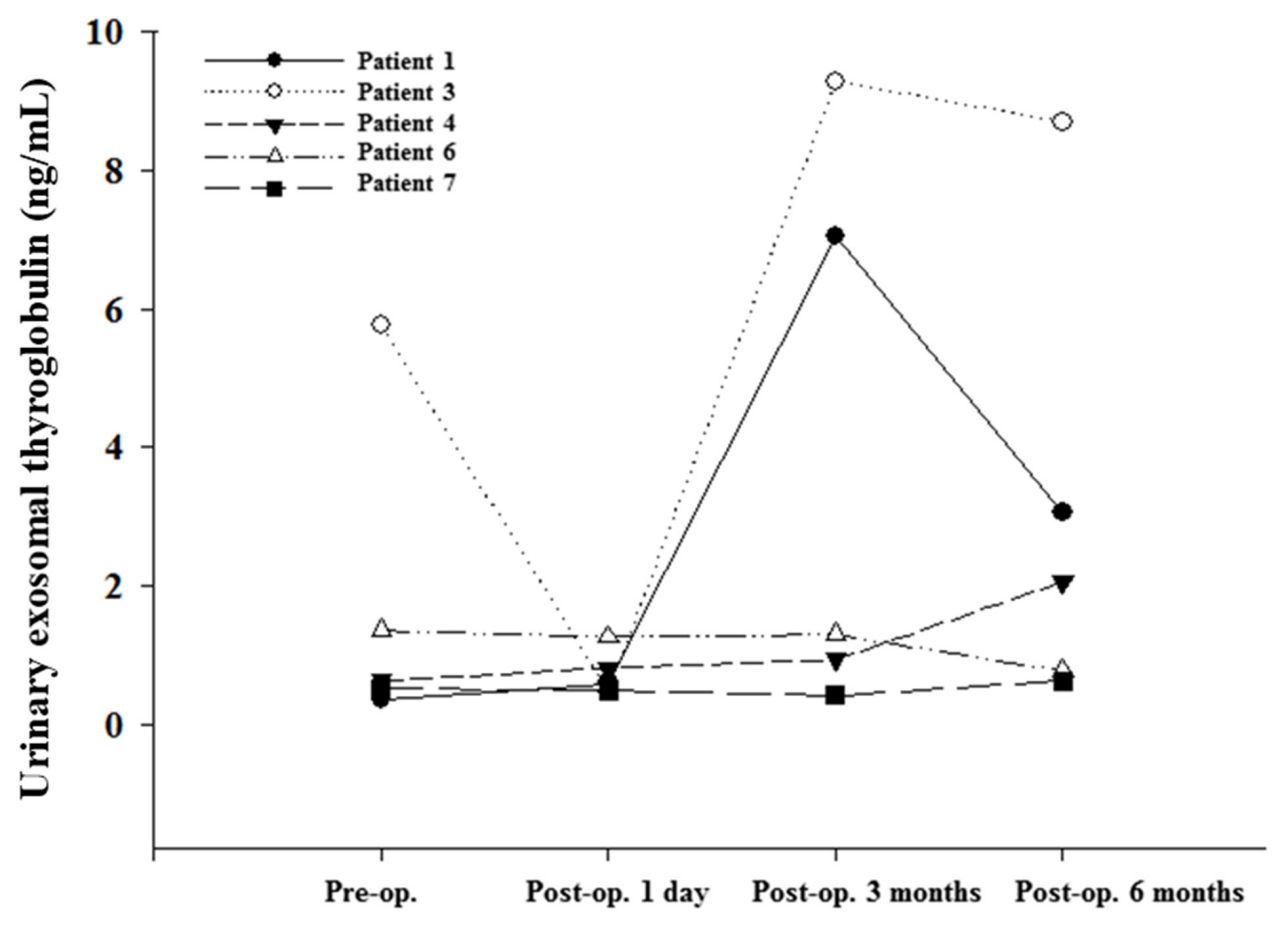
Urinary exosomal thyroglobulin showed an increasing trend in patients of post thyroidectomy and/or I-131 ablation in comparison with serum thyroglobulin.

We classified U-Ex Tg level according to the thyroid cancer stage (Figure 1), fourteen patients (patient 3 and 9 were excluded due to distant metastasis or suspect delphian lymph node involvement) revealed that U-Ex Tg was statically significant higher in larger tumor, which point out that U-Ex Tg can implicit the tumor burden. In addition, pre-operative and post-operative serial changes of U-Ex Tg in patients receiving total thyroidectomy and/or radio-active iodine ablation are revealed in Figure 2. The U-Ex Tg levels still showed trends of elevation in patients 1, 3, and 4, who were papillary thyroid cancer with soft tissue involvement or metastasis of level IV lymph nodes. However, serum thyroglobulin cannot be detected in such patients after ablative therapy.

We hope to emphasize the difference of U-Ex Tg between various surgical findings. The pathological findings of patients 1, 3, 4 revealed larger tumor (T3) or lymph nodes metastasis, in contrast to patients 6 and 7 with smaller tumor without lymph nodes metastasis. In addition, patient 1, 3, and 4 received ablative I-131 treatment without detectable serum thyroglobulin, but prominently elevated U-Ex Tg postoperatively. However, patient 6, who did not receive ablative I-131, was noted of detectable serum thyroglobulin, but very lower U-Ex Tg. The difference between these two groups revealed that U-Ex Tg will be an earlier and brand-new biomarker to predict the prognosis or recurrence for patients with DTC post-operatively.

## Discussion

Our study is the first study to evaluate whether urinary exosomal thyroglobulin (U-Ex Tg) is a reliable biological marker as a substitute for serum thyroglobulin. Such patients are not required to withdraw thyroid hormone or receive rhTSH stimulation. For patients with thyroid cancer who received thyroidectomy with or without ablative radioactive I-131 therapy, serum thyroglobulin is defined as a cancer biomarker during follow-up (4–8). If thyroglobulin cannot be detected in the serum, patients are considered to have completed treatment, independently of the interference of anti-thyroglobulin antibody (4). Typically, serum thyroglobulin cannot be detected even under costly rhTSH stimulation in patients with biochemically complete treatment. Therefore, currently no serial biomarkers are available for evaluating and predicting cancer recurrence.

Exosomes are membrane-derived extracellular vesicles with size about 40–100 nm, which are found in consistent concentrations in all body fluids, including blood, saliva and urine. In this study, U-Ex Tg was shown to be a non-invasive, reproducible, convenient, serial, and accurate follow-up marker for patients with thyroid cancer, as we used peptide sequences to quantify the levels of thyroglobulin in urine exosomes. Without the requirement for rhTSH, costs are reduced, and patients can continue the use of thyroid hormone during cancer follow-up. We also used galectin-3 for comparison to confirm the trends in thyroglobulin levels. Galectin-3 modulates cell growth via galactosidase-binding protein, which is correlated with occurrence and metastasis of papillary thyroid carcinoma (14–16). In this study, the trends in the changes of U-Ex Tg to be higher in patients with extra-thyroid invasion, metastasis of neck level IV lymph nodes, and lymph-vascular invasion. While the patients received ablation of radioactive I-131 therapy six months later, serum thyroglobulin was not detected. However, the increasing tendency of U-Ex Tg suggests that probable recurrence.

In recent years, progress in peptide mass spectrometry methods has provided a cost-effective, accurate method for identifying biomarkers. Large profiling of proteomics of human urine reveled different follow-up manners (17, 18). From the perspective of oncology, one option is to identify new biomarkers for earlier diagnosis of various cancers, while another option is to prevent follow-up residual tumor formation and cancer recurrence. The goal of our study is to identify a new pathway for tracking biomarkers in patients with thyroid cancer receiving ablative surgery and radioactive I-131 treatment. Exosomal proteins can influence cellular signaling, inflammation, immunity. In addition, inflammatory exosomal proteins contribute to various patho-physiological processes in cellular behavior (19). Compared with serum thyroglobulin, U-Ex Tg can be an important pro-inflammatory predictor and biomarker of thyroid cancer recurrence for certain patients. For patients receiving total thyroidectomy and/or ablation with radioactive iodine, the increasing tendency of U-Ex Tg revealed that it can be used as a substitute for undetectable serum thyroglobulin in predicting the recurrence of thyroid cancer. U-Ex Tg could be an important alternative to serum thyroglobulin which advantages technology and eases requirements for patients. Our limitation is limited current patient numbers, but the study is still ongoing with a larger cohort in patients of thyroid cancer, who received thyroidectomy and/or radioactive iodine at least one year.

In our study, the most important and useful scenario in using a test that is potentially more sensitive than existing serological testing is to eliminate the suspicion of recurrence and remove subjects from long term follow up.

## Strengths and Limitations of this Study

1. The aim of the present study is to analyze the urinary exosomal proteins to identify early prognostic biological markers in the urine in this prospective study.
2. The significant and novel finding in the present study is that in comparison with serum thyroglobulin, urinary exosomal thyroglobulin (U-Ex Tg) can be an important pro-inflammatory predictor and biomarker of thyroid cancer recurrence. U-Ex Tg could be an important alternative to serum thyroglobulin which advantages technology and eases requirements for patients.
3. Current patient numbers are limited, but the study is still ongoing to collect more data from urinary exosomal proteins of patients with thyroid cancer.

## Data Availability Statement

The datasets presented in this article are not readily available because the study is still ongoing with a larger cohort in patients of thyroid cancer. Requests to access the datasets should be directed to Chih-Yuan Wang: cyw1965@gmail.com.

## Ethics Statement

The studies involving human participants were reviewed and approved by Institution Review Board of National Taiwan University Hospital. The patients/participants provided their written informed consent to participate in this study. Written informed consent was obtained from the individual(s) for the publication of any potentially identifiable images or data included in this article.

## Author's Note

This study had accepted as a poster presentation at the 88th Annual Meeting of the American Thyroid Association, 2018; Poster ID: 409. https://www.liebertpub.com/doi/10.1089/thy.2018.29065.abstracts.

## Author Contributions

C-YW: conception and design and development of methodology. T-YH, C-YW, K-YC, and L-TH: acquisition of data (provided animals, acquired and managed patients, provided facilities, etc.). T-YH, C-YW, and L-TH: analysis and interpretation of data (e.g., statistical analysis, biostatistics, computational analysis). T-YH and C-YW: writing, review, and/or revision of the manuscript. C-YW and L-TH: administrative, technical, or material support (i.e., reporting or organizing data, constructing databases), Other (application to regional ethics committee), and Other (supply of reagents generated by herself). C-YW and K-YC: study supervision. All authors contributed to the article and approved the submitted version.

## Conflict of Interest

The authors declare that the research was conducted in the absence of any commercial or financial relationships that could be construed as a potential conflict of interest.
