# Supplementary material for: Urinary Exosomal Thyroglobulin in Thyroid Cancer Patients With Post-ablative Therapy: A New Biomarker in Thyroid Cancer
Source: Front Endocrinol (Lausanne). 2020 Jun 16;11:382. doi: 10.3389/fendo.2020.00382 (PMC7308545; doi:10.3389/fendo.2020.00382)

Supplemental legends:

Table 1: Peptide concentrations of thyroglobulin and galectin-3 reveal as μM

Table 2: Peptide concentrations of thyroglobulin and galectin-3 reveal as ng/mL

Figure 1-7: Trends of urinary peptide biomarkers (thyroglobulin and galectin-3) concentrations in time-dependent changes during six months in patients with thyroid ablation (Patient 1-7)

Supplemental data

Table 1 Peptide concentrations (µM)

	Peptide	Pre-op.	Post-op. 1 day	Post-op. 3 months	Post-op. 6 months
**Patient 1**	**Thyroglobulin**	0.0003	0.0005	0.0060	0.0026
	**Galectin-3**	0.0018	0.0025	0.0030	0.0042
**Patient 2**	**Thyroglobulin**	0.0016	0.0065	0.0030	0.0005
	**Galectin-3**	0.0022	0.0045	0.0017	0.0007
**Patient 3**	**Thyroglobulin**	0.0049	0.0004	0.0079	0.0074
	**Galectin-3**	0.0033	0.0012	0.0021	0.0051
**Patient 4**	**Thyroglobulin**	0.00053	0.00069	0.00079	0.00175
	**Galectin-3**	0.00086	0.00093	0.00156	0.00139
**Patient 5**	**Thyroglobulin**	0.00080	0.00054	0.00056	0.00056
	**Galectin-3**	0.00143	0.00090	0.00115	0.00121
**Patient 6**	**Thyroglobulin**	0.00115	0.00107	0.00110	0.00065
	**Galectin-3**	0.00153	0.00129	0.00123	0.00090
**Patient 7**	**Thyroglobulin**	0.00044	0.00041	0.00035	0.00058
	**Galectin-3**	0.00088	0.00083	0.00083	0.00109

Supplemental data

Table 2 Peptide concentrations (ng/mL)

	Peptide	Pre-op.	Post-op. 1 day	Post-op. 3 months	Post-op. 6 months
**Patient 1**	**Thyroglobulin**	0.35291	0.58819	7.06	3.06
	**Galectin-3**	1.55	2.16	2.59	3.62
**Patient 2**	**Thyroglobulin**	1.88	7.65	3.53	0.58819
	**Galectin-3**	1.9	3.88	1.47	0.6034
**Patient 3**	**Thyroglobulin**	5.76	0.47055	9.29	8.71
	**Galectin-3**	2.84	1.03	1.81	4.4
**Patient 4**	**Thyroglobulin**	0.623	0.812	0.929	2.059
	**Galectin-3**	0.741	0.802	1.345	1.198
**Patient 5**	**Thyroglobulin**	0.941	0.635	0.659	0.659
	**Galectin-3**	1.233	0.776	0.991	1.043
**Patient 6**	**Thyroglobulin**	1.353	1.259	1.294	0.765
	**Galectin-3**	1.319	1.112	1.060	0.776
**Patient 7**	**Thyroglobulin**	0.518	0.482	0.412	0.682
	**Galectin-3**	0.759	0.715	0.715	0.940

Supplemental data

Figure 1 Trends of urinary peptide biomarkers concentrations (Patient 1)

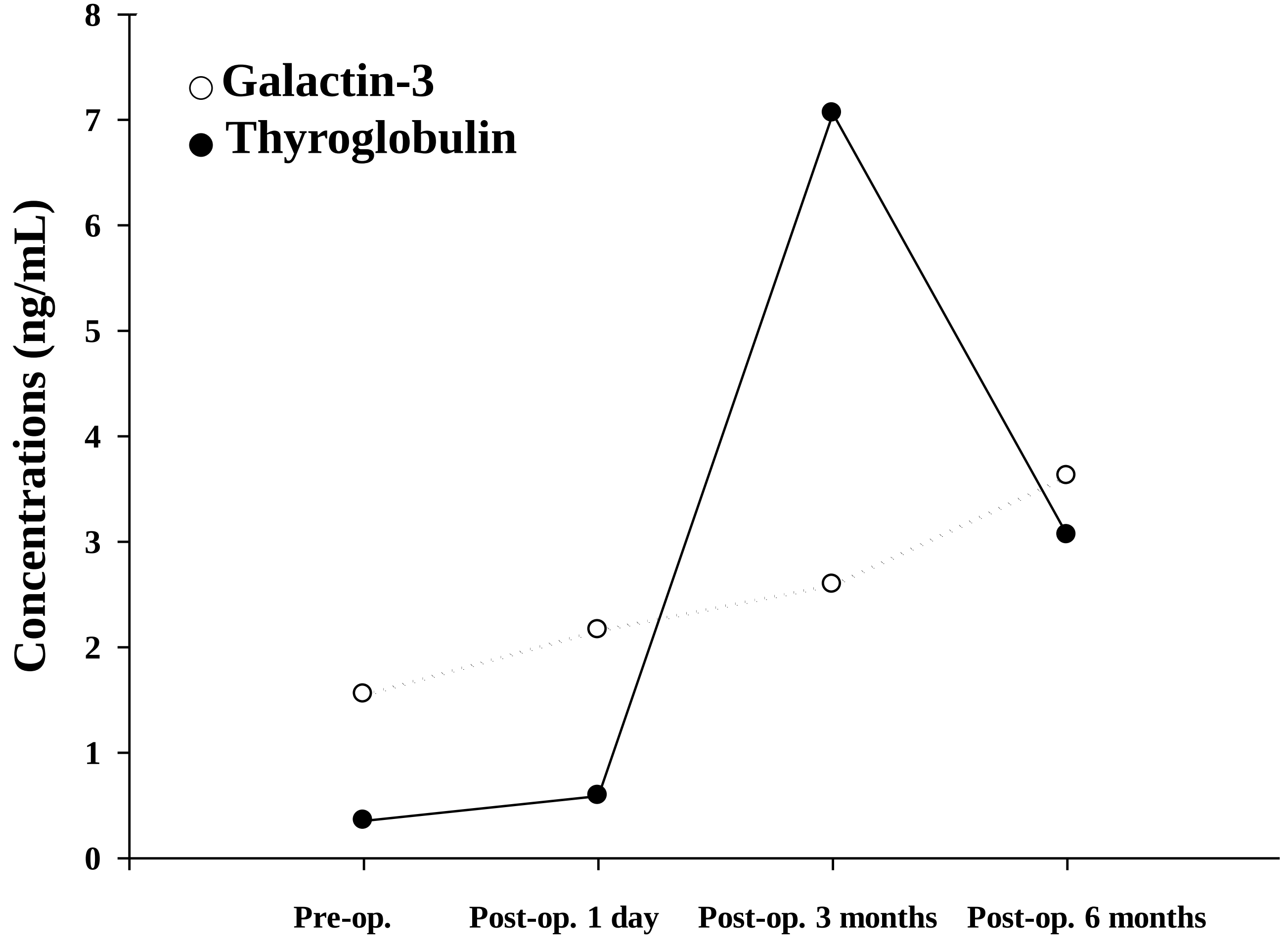

Supplemental data

Figure 2 Trends of urinary peptide biomarkers concentrations (Patient 2)

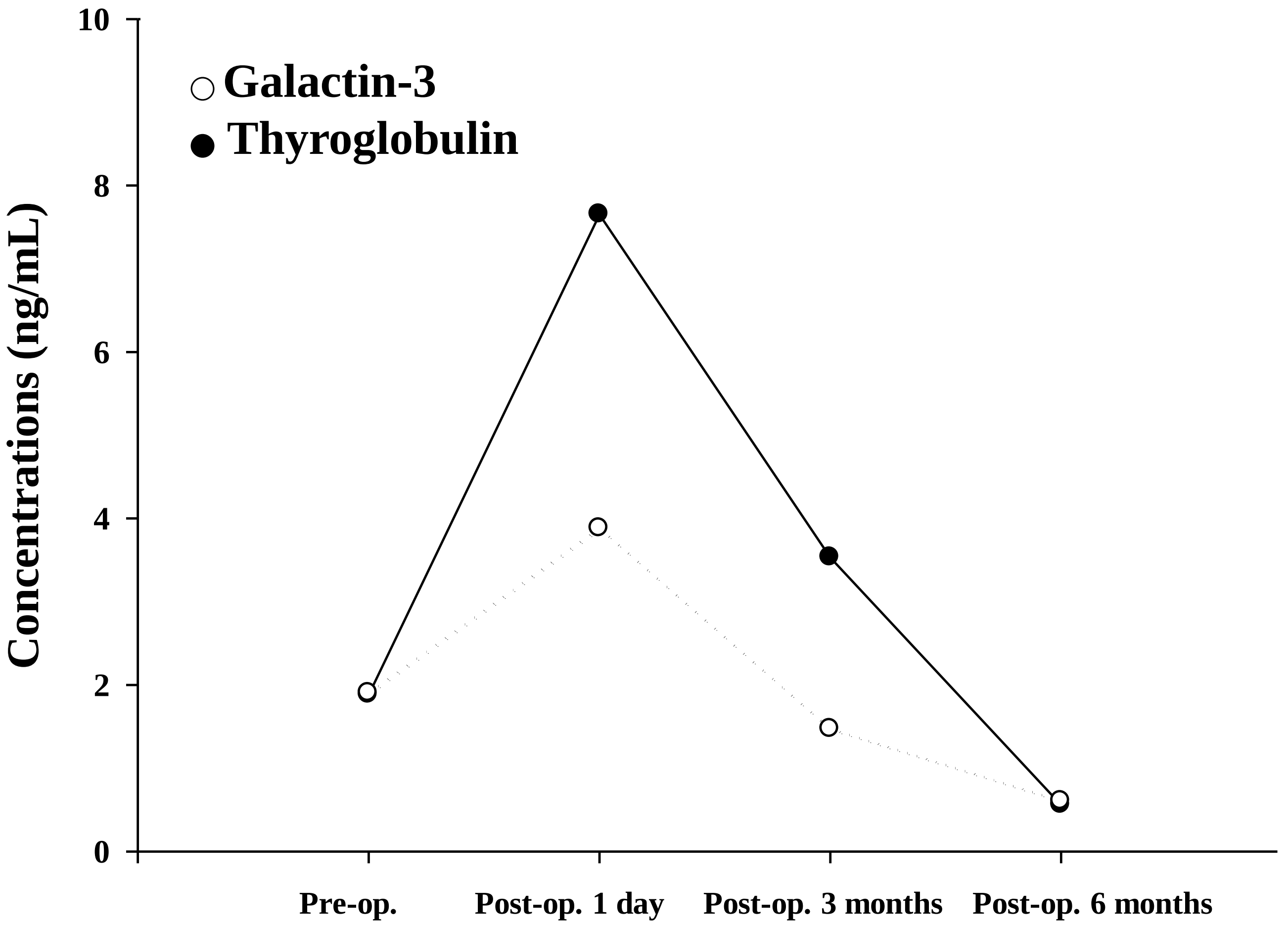

Supplemental data

Figure 3 Trends of urinary peptide biomarkers concentrations (Patient 3)

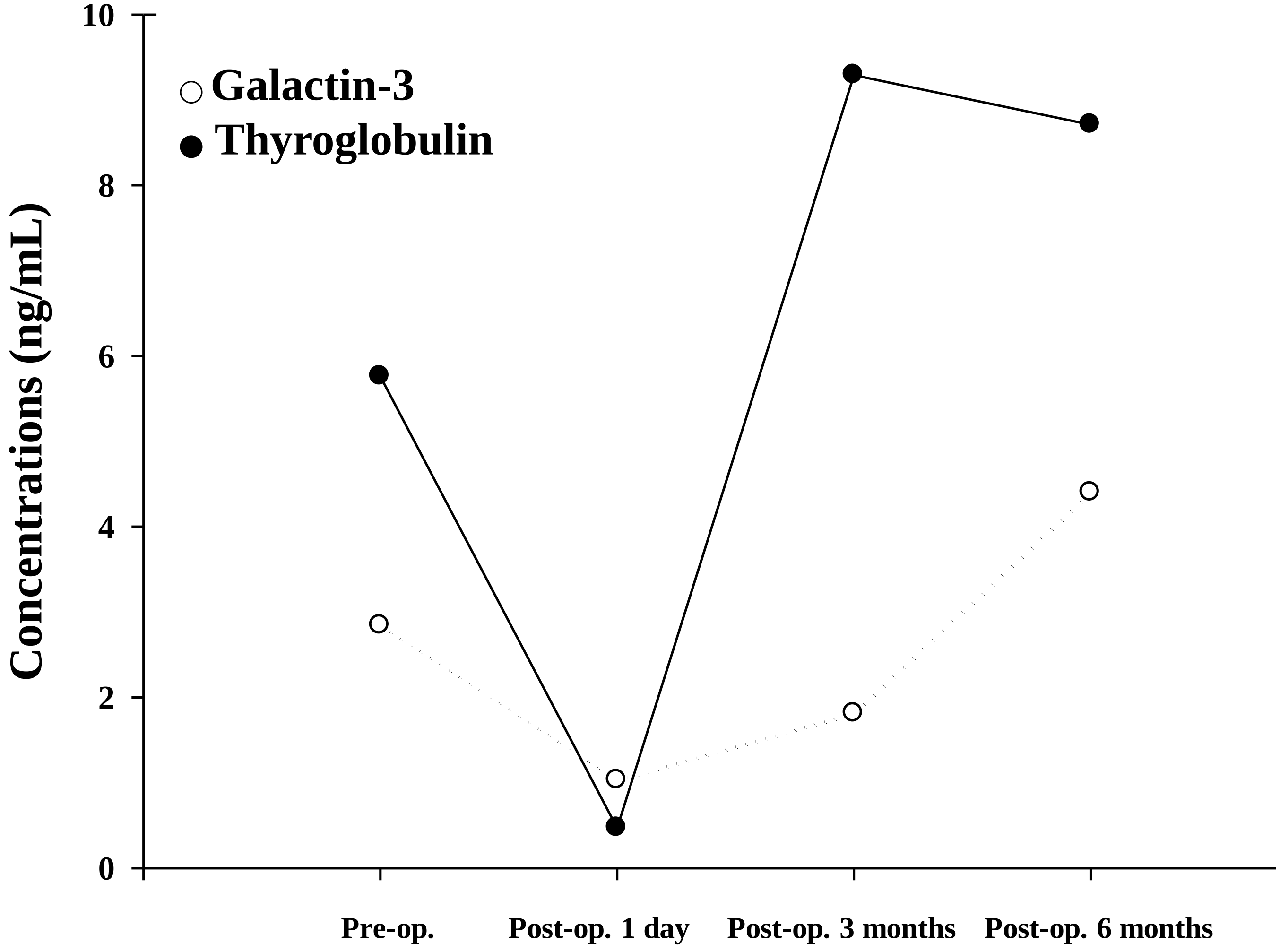

Supplemental data

Figure 4 Trends of urinary peptide biomarkers concentrations (Patient 4)

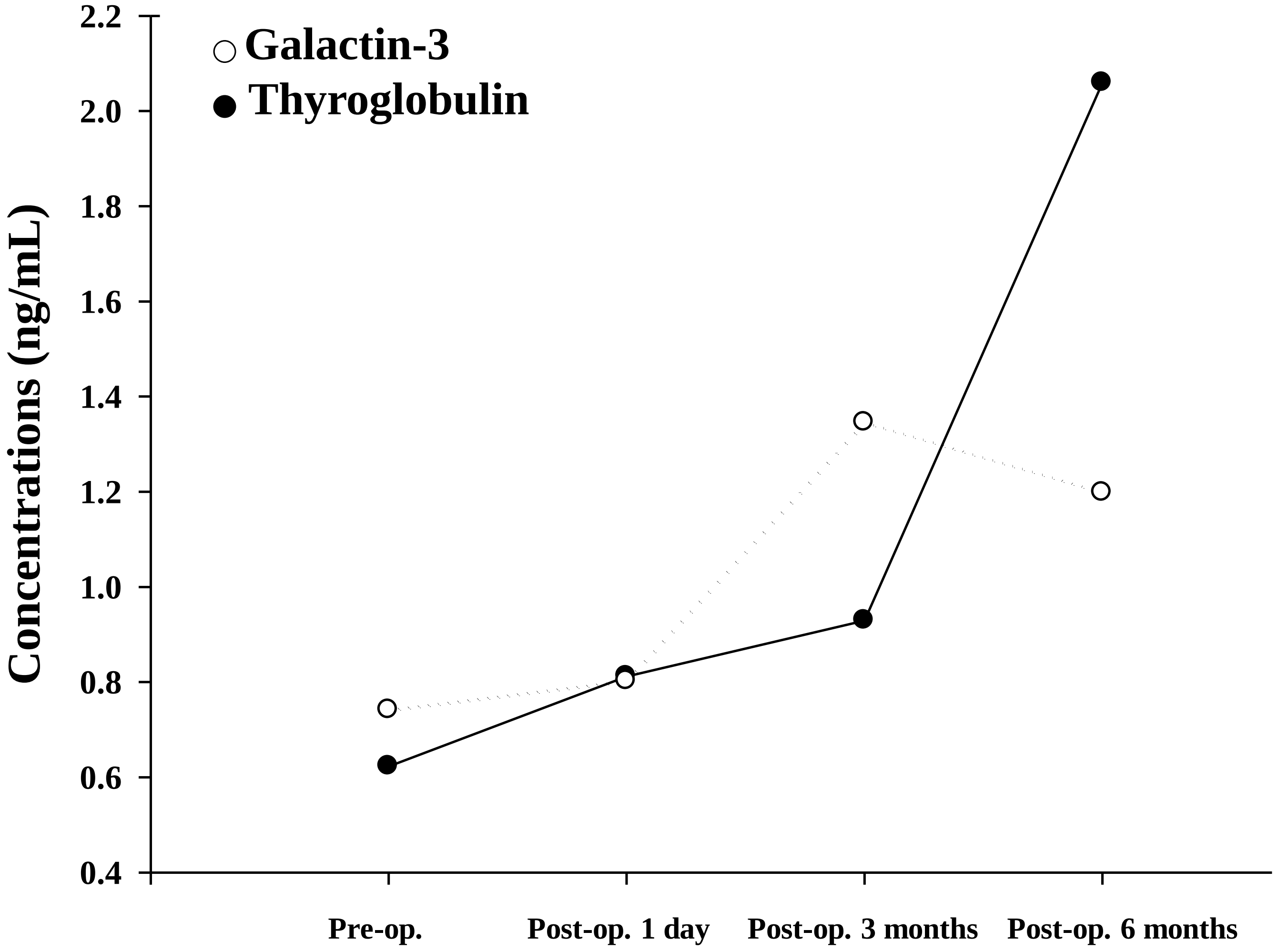

Supplemental data

Figure 5 Trends of urinary peptide biomarkers concentrations (Patient 5)

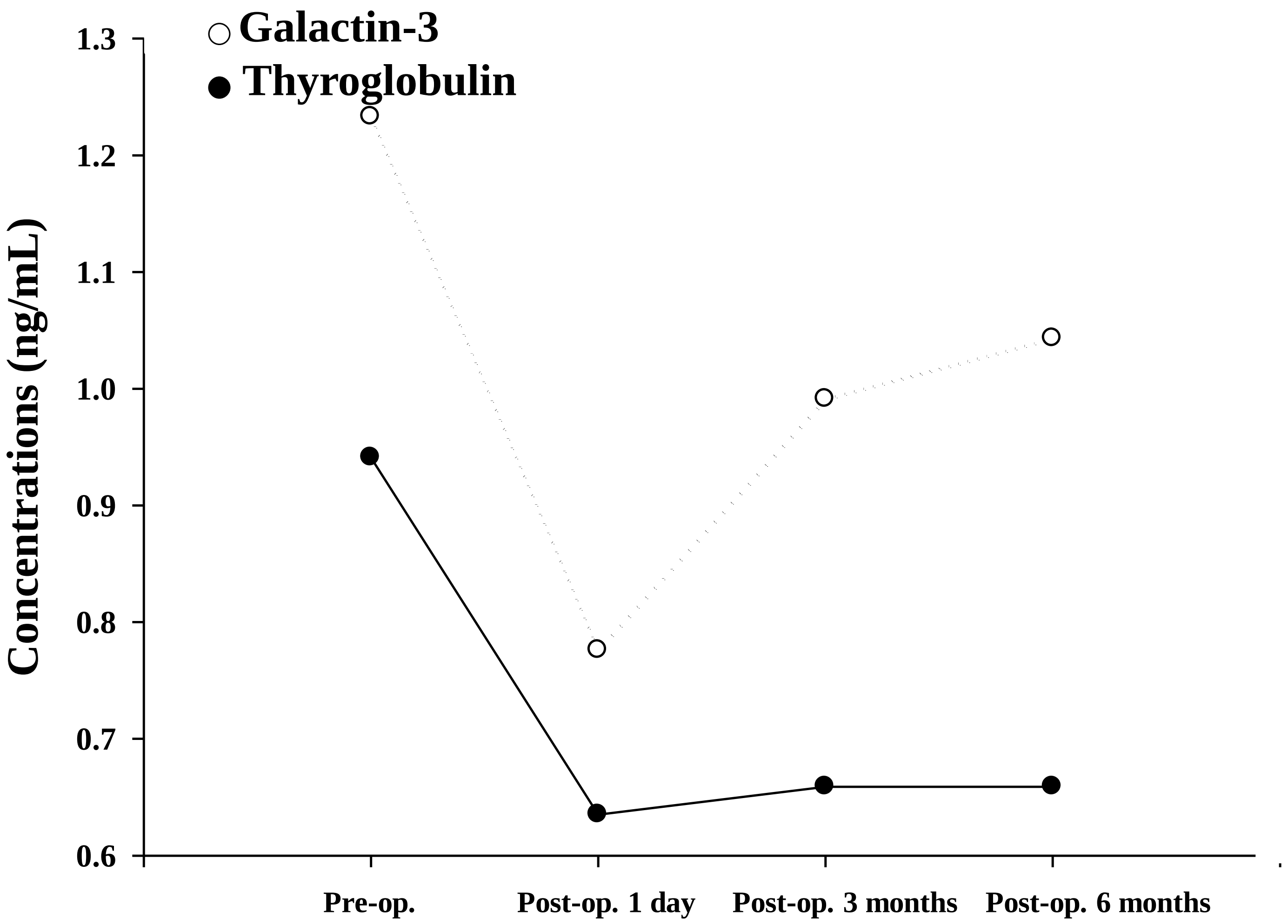

Supplemental data

Figure 6 Trends of urinary peptide biomarkers concentrations (Patient 6)

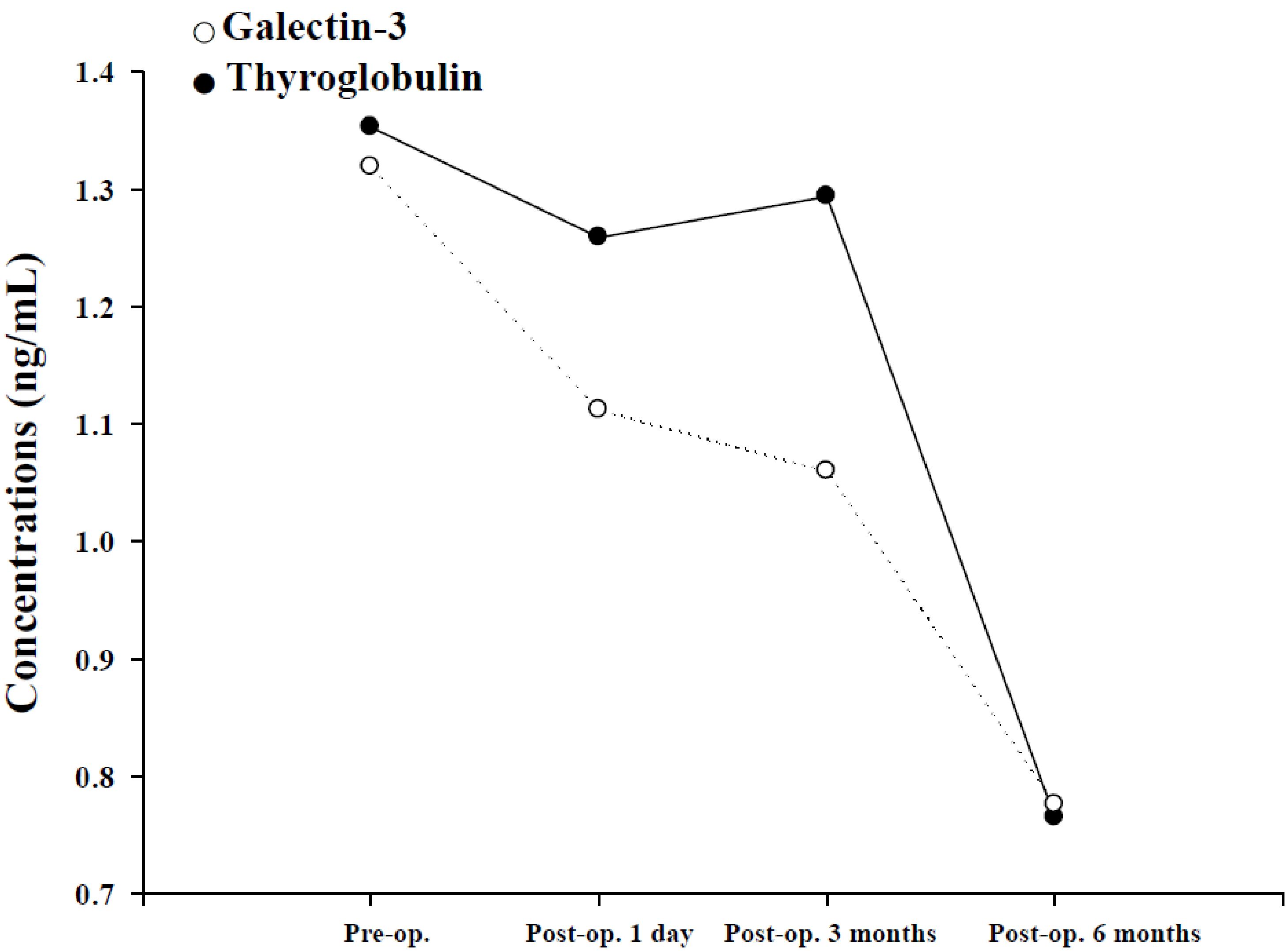

Supplemental data

Figure 7 Trends of urinary peptide biomarkers concentrations (Patient 7)

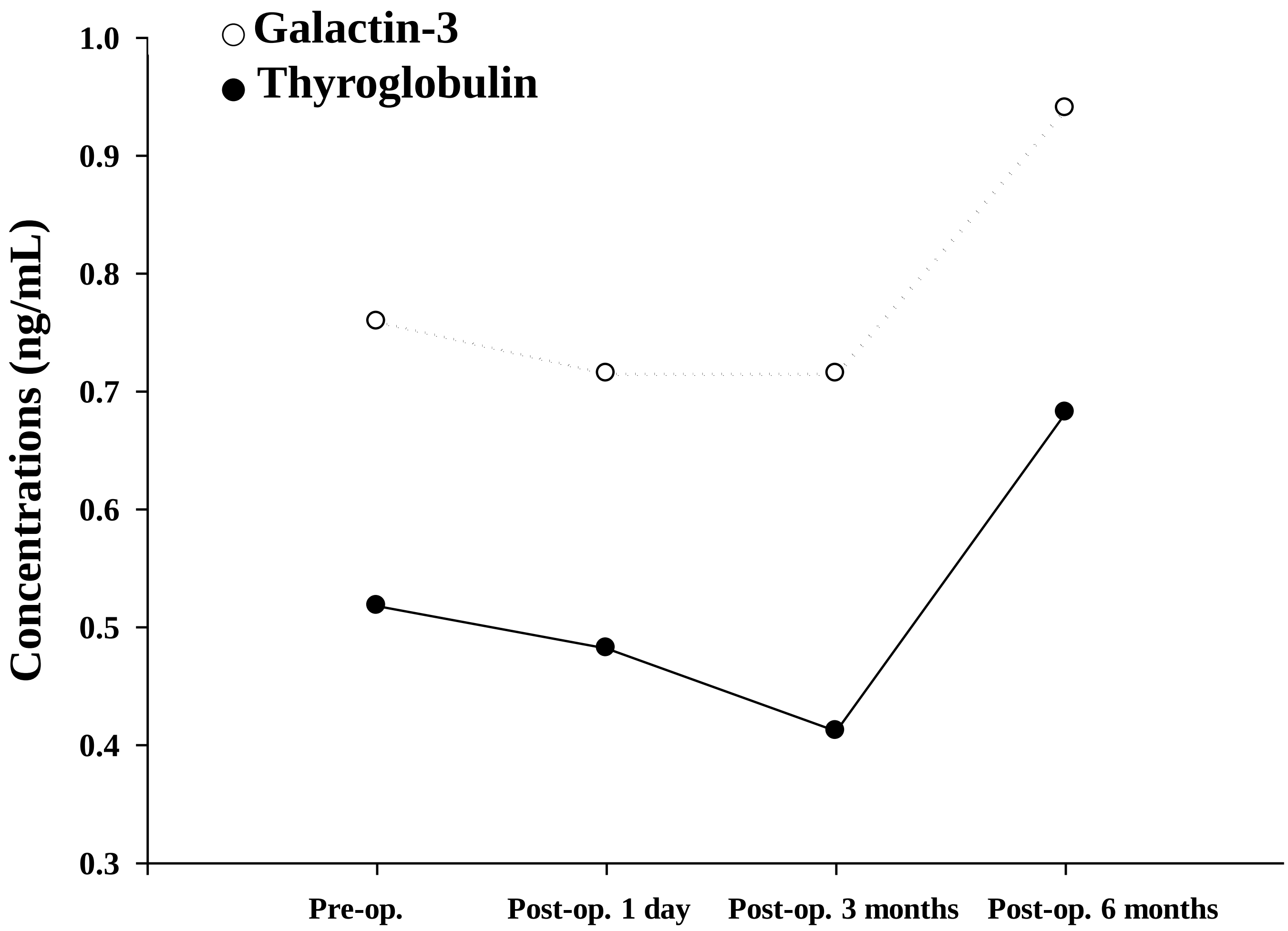

Supplement: Supplementary file 1 [file Data_Sheet_1.PDF]
